# Developmental Neurotoxicity of Perfluorinated Chemicals Modeled *in Vitro*

**DOI:** 10.1289/ehp.11253

**Published:** 2008-03-03

**Authors:** Theodore A. Slotkin, Emiko A. MacKillop, Ronald L. Melnick, Kristina A. Thayer, Frederic J. Seidler

**Affiliations:** 1 Department of Pharmacology and Cancer Biology, Duke University Medical Center, Durham, North Carolina, USA; 2 National Toxicology Program, National Institute of Environmental Health Sciences, National Institutes of Health, Department of Health and Human Services, Research Triangle Park, North Carolina, USA

**Keywords:** developmental neurotoxicity, PC12 cells, perfluorinated chemicals, perfluoroalkyl acids, perfluorobutane sulfonate, perfluorooctane sulfonamide, perfluorooctane sulfonate, perfluorooctanoate, perfluorooctanoic acid

## Abstract

**Background:**

The widespread detection of perfluoroalkyl acids and their derivatives in wildlife and humans, and their entry into the immature brain, raise increasing concern about whether these agents might be developmental neurotoxicants.

**Objectives:**

We evaluated perfluorooctane sulfonate (PFOS), perfluorooctanoic acid (PFOA), perfluorooctane sulfonamide (PFOSA), and perfluorobutane sulfonate (PFBS) in undifferentiated and differentiating PC12 cells, a neuronotypic line used to characterize neurotoxicity.

**Methods:**

We assessed inhibition of DNA synthesis, deficits in cell numbers and growth, oxidative stress, reduced cell viability, and shifts in differentiation toward or away from the dopamine (DA) and acetylcholine (ACh) neurotransmitter phenotypes.

**Results:**

In general, the rank order of adverse effects was PFOSA > PFOS > PFBS ≈ PFOA. However, superimposed on this scheme, the various agents differed in their underlying mechanisms and specific outcomes. Notably, PFOS promoted differentiation into the ACh phenotype at the expense of the DA phenotype, PFBS suppressed differentiation of both phenotypes, PFOSA enhanced differentiation of both, and PFOA had little or no effect on phenotypic specification.

**Conclusions:**

These findings indicate that all perfluorinated chemicals are not the same in their impact on neurodevelopment and that it is unlikely that there is one simple, shared mechanism by which they all produce their effects. Our results reinforce the potential for *in vitro* models to aid in the rapid and cost-effective screening for comparative effects among different chemicals in the same class and in relation to known developmental neurotoxicants.

Perfluoroalkyl acids (PFAAs) are in use as surfactants; also, they are formed as breakdown products of larger polymers in industrial use, and they accumulate in the environment because of their chemical stability and general lack of biodegradation (Lau et al. 2004, 2006, 2007). There is increasing concern over the body burdens of these agents in both wildlife and humans, driven not only by their production and release into the environment but also because of their extremely long biologic retention times (D’Eon et al. 2006; D’Eon and Mabury 2007; Lau et al. 2004, 2006, 2007; Schroder 2003; Schultz et al. 2006; Xu et al. 2004, 2006). For example, the human half-lives for two of the most prevalent PFAAs, perfluorooctane sulfonate (PFOS) and perfluorooctanoic acid (PFOA), are in the range of 4–6 years (Lau et al. 2007; Olsen et al. 2007). Serum levels in production workers typically average 0.5–2 μg/mL, with the highest reported values reaching 13 μg/mL (26 μM) for PFOS and 114 μg/mL (276 μM) for PFOA (Lau et al. 2007; Olsen et al. 1998, 1999, 2003a, 2003b). In the general population, average values recorded in 2003–2004 were about 20 ng/mL for PFOS and 4 ng/mL for PFOA, down by about one-third from those reported in 1999–2000 (Calafat et al. 2007a, 2007b). North American wildlife populations now show values well into the tens of micromolar range (Lau et al. 2007). Although 3M (St. Paul, MN) has phased out production of PFOS, and PFOA and PFOA precursor compounds have been committed to a voluntary phase-out (U.S. Environmental Protection Agency 2006), the resistance of these compounds to degradation will result in their persistence in the environment for many years. Further, the newer fluorochemicals, such as perfluorobutane sulfonic acid (PFBS), which were introduced as replacement for PFOS or PFOA because of their shorter half-lives in people (3M 2007; Lau et al. 2007), need to be evaluated for potential common mechanisms of action.

Despite the highly polar nature of their sulfonyl or carboxyl head groups, PFOS and PFOA enter the developing brain, possibly reflecting either their surfactant properties or the immaturity of the fetal/neonatal blood–brain barrier (Lau et al. 2004, 2006, 2007). Accordingly, these agents need to be evaluated for their potential to elicit developmental neurotoxicity. Developmental toxicity in rodents given high doses of PFOS leads to neonatal morbidity and mortality but also elicits neurologic delays (Lau et al. 2003; Luebker et al. 2005a, 2005b). Recently, Johansson et al. (2008) found that much lower developmental exposures of mice to PFOS and PFOA produced behavioral defects persisting into adulthood, specifically involving acetylcholine (ACh) systems, as inferred from altered responses to nicotine; this suggests that brain development is indeed affected at exposures below those required for systemic toxicity. Nevertheless, these studies cannot distinguish whether the neurodevelopmental effects of the perfluorinated chemicals represent direct neurotoxic mechanisms as opposed to indirect consequences of antithyroid actions, effects on peroxisome proliferator receptor-α, effects on maternal–fetal or maternal–neonatal physiology or behavior, neonatal hypoxic episodes from compromised respiratory function (as reported at higher exposures), or any of the myriad possibilities from cryptic or overt systemic toxicity (Lau et al. 2004, 2006, 2007). One approach to identify direct developmental neurotoxicity is to use an *in vitro* model, where these confounding factors do not operate. In one study, PFOS was shown to impair cerebellar Purkinje cell function *in vitro*; although this did not involve protracted effects or neurodevelopment, it pointed out the potential for direct actions of perfluorinated chemicals on neuronal cells (Harada et al. 2006).

In the present study, we used PC12 cells, a standard *in vitro* model for neuronal development (Teng and Greene 1994) that has already been used to characterize essential features of the developmental neurotoxicity of diverse compounds such as organophosphate and carbamate pesticides, organochlorines, metals, neuroactive drugs, oxidative stressors, and a host of other agents (Bagchi et al. 1995; Costa 1998; Crumpton et al. 2000b, 2001; Flaskos et al. 1994; Li et al. 1999; Qiao et al. 2003; Ramesh et al. 1999; Slotkin et al. 2007; Song et al. 1998; Tuler et al. 1989). As transformed cells, the PC12 line has an advantage over cultured primary neurons, which do not maintain cell division and thus cannot detect adverse effects on the cell cycle, a likely neurotoxic target. Furthermore, primary neurons do not provide a uniform population either in terms of cell types or differentiation state, rendering their use for identification of direct neurotoxic mechanisms problematic. Upon exposure to nerve growth factor, PC12 cells gradually exit the mitotic cycle and begin to differentiate, developing axonal projections, electrical excitability, and two distinct neurotransmitter phenotypes, ACh and dopamine (DA) (Fujita et al. 1989; Song et al. 1998; Teng and Greene 1994); this renders them particularly suitable for examining whether the effects of PFOS and PFOA on ACh systems reported *in vivo* (Johansson et al. 2008) are likely to reflect direct neurotoxic actions. Nevertheless, PC12 cells share the limitations common to *in vitro* models, namely, difficulty in modeling neuronal–glial or other cell-to-cell interactions, or architectural aspects of regional development, maternal–fetal or neonatal pharmacokinetics, and related issues of bioavailability, dose, and bioeffective concentrations (Costa 1998; Slotkin 2004b).

We focused on four perfluorinated chemicals: PFOS and PFOA, the agents for which bioaccumulation is currently the highest; perfluorooctane sulfonamide (PFOSA), a potent mitochondrial toxicant (Starkov and Wallace 2002) and less polar precursor to PFOS, found in human tissues; and PFBS, a representative of the newer PFAAs with much shorter biologic half-lives (Lau et al. 2007). The design of an appropriate concentration range for *in vitro* studies is somewhat arbitrary, given that we are using a transformed cell line and that exposures occur over a very short period (hours to days) as opposed to much longer exposures *in vivo* (e.g., throughout gestation, infancy, or childhood). We chose to evaluate concentrations of 10–250 μM, lying in the upper range of human serum levels (Lau et al. 2007; Olsen et al. 1998, 1999, 2003a, 2003b). We also included a positive test substance for comparison with the perfluorinated chemicals, as recommended for developmental neurotoxicity testing so as to demonstrate the capacity to identify significant effects, as well as to provide a benchmark for comparison to a known developmental toxicant (Crofton et al., in press). For our purposes, we used chlorpyrifos (CPF), an organophosphate pesticide whose developmental neurotoxicity has been well-characterized both *in vivo* and *in vitro* (Slotkin 1999, 2004a, 2005) and for which the PC12 model recapitulates the underlying cellular mechanisms that operate in the intact, developing brain (Bagchi et al. 1995; Crumpton et al. 2000a; Das and Barone 1999; Jameson et al. 2006b, 2007; Qiao et al. 2001; Slotkin et al. 2007; Song et al. 1998). Our evaluations were conducted for cells in both the undifferentiated state and during differentiation, focusing on indices of cell replication (radiolabeled thymidine incorporation into DNA), cell number (amount of DNA in the culture), cell growth (total protein/DNA ratio, membrane/total protein ratio), viability (trypan blue exclusion), and phenotype (DA vs. ACh). Each neural cell contains only a single nucleus (Winick and Noble 1965), so the DNA content (micrograms of DNA per culture dish in the present study) reflects the total number of cells (Song et al. 1998). Indices of growth were provided by measurements of protein subfractions related to cell size and membrane surface area (Jameson et al. 2006a; Thai et al. 1996). The total protein/DNA ratio rises with cell enlargement and, with the onset of differentiation, the development of neuritic projections necessitates a rise in the relative contribution of membrane proteins, so the increase in the membrane/total protein ratio gives an indication of augmented membrane “complexity.” The effects on cell number, size, and cell surface area were compared to those on viability, evaluated by trypan blue exclusion, and lipid peroxidation, determined from the formation of malondialdehyde (MDA). To characterize the DA and ACh phenotypes, we assessed the activities of the biosynthetic enzymes for these two neurotransmitters, tyrosine hydroxylase (TH) and choline acetyltransferase (ChAT), respectively (Teng and Greene 1994).

## Materials and Methods

All of the techniques used in this study have been reported previously; thus, only brief descriptions are given here.

### Cell cultures

Because of the clonal instability of the PC12 cell line (Fujita et al. 1989), the experiments were performed on cells that had undergone fewer than five passages. PC12 cells (American Type Culture Collection, 1721-CRL; obtained from the Duke Comprehensive Cancer Center, Durham, NC) were grown under standard conditions (Crumpton et al. 2000a; Qiao et al. 2003; Song et al. 1998) in RPMI-1640 medium (Invitrogen, Carlsbad, CA) supplemented with 10% inactivated horse serum (Sigma Chemical Co., St. Louis, MO), 5% fetal bovine serum (Sigma), and 50 μg/mL penicillin streptomycin (Invitrogen); cells were incubated with 7.5% CO_2_ at 37°C. For studies in the undifferentiated state, the medium was changed 24 hr after seeding to include 50 μM CPF (98.8% purity; Chem Service, West Chester, PA), or varying concentrations of each of the four perfluorinated chemicals (supplied by Battelle, Columbus, OH): PFOS (97% purity), PFOA (99.2% purity), PFOSA (99.4% purity), and PFBS potassium salt (98.2% purity). Because of the limited water solubility of CPF and some of the perfluorinated chemicals, all test agents were dissolved in dimethyl sulfoxide (DMSO) to achieve a final concentration in the culture medium of 0.1%, which has no effect on replication or differentiation of PC12 cells (Qiao et al. 2001, 2003; Song et al. 1998); control cultures contained the same concentration of DMSO. For studies in differentiating cells, 24 hr after seeding, the medium was changed to include 50 ng/mL of 2.5 S murine nerve growth factor (Invitrogen) and DMSO with or without the test agents; these cells were examined for up to 6 days, with medium changes (including test agents) every 48 hr. We chose the CPF concentration to elicit a robust response for each of the effects to be compared to the actions of perfluorinated chemicals, and accordingly, we selected a concentration that elicits inhibition of DNA synthesis and interference with cell acquisition and oxidative stress, but that lies below the threshold for outright cytotoxicity or loss of viability (Bagchi et al. 1995; Crumpton et al. 2000b; Das and Barone 1999; Jameson et al. 2006b; Qiao et al. 2001, 2003, 2005; Slotkin et al. 2007; Song et al. 1998).

### DNA synthesis

We measured DNA synthesis in undifferentiated cells. Twenty-four hours after plating, we changed the medium to include the test agents. After 23 hr of exposure, we initiated the measurement of DNA synthesis by changing the medium to include 1 μCi/mL of [^3^H]thymidine (specific activity, 2 Ci/mmol; GE Healthcare, Piscataway, NJ) along with the continued inclusion of the test substances. After 1 hr, the medium was aspirated and cells were harvested, and DNA was precipitated and separated from other macromolecules by established procedures that produce quantitative recovery of DNA (Bell et al. 1986; Slotkin et al. 1984). The DNA fraction was counted for radiolabel and the DNA concentration was determined spectrophotometrically by absorbance at 260 nm. We corrected incorporation values to the amount of DNA present in each culture to provide an index of DNA synthesis per cell (Winick and Noble 1965), and we recorded the total DNA content.

### DNA and protein ratios

We determined DNA and protein ratios in differentiating cells after 6 days of continuous exposure to the test agents. Cells were harvested and washed, and the DNA and protein fractions were isolated and analyzed as described previously (Slotkin et al. 2007; Song et al. 1998), with DNA and total protein analyzed by dye binding (Trauth et al. 2000). To prepare the cell membrane fraction, the homogenates were sedimented at 40,000 × *g* for 10 min and the pellet was washed and resedimented. Aliquots of the final resuspension were then assayed for membrane protein (Smith et al. 1985).

### Oxidative stress

We evaluated the degree of lipid peroxidation in undifferentiated cells after 24 hr of exposure to test agents, and in differentiating cells after 4 days of exposure. We measured the concentration of MDA by reaction with thiobarbituric acid using a modification (Qiao et al. 2005) of published procedures (Guan et al. 2003). To give the MDA concentration per cell, values were calculated relative to the amount of DNA.

### Viability

To assess cell viability, the cell culture medium was changed to include trypan blue (1 volume per 2.5 vol of medium; Sigma) and cells were examined for staining under 400× magnification, counting an average of 100 cells per field in four different fields per culture. Assessments were made after 24 hr of exposure in undifferentiated cells and after 4 days for differentiating cells.

### Enzyme activities

Differentiating cells were harvested after 6 days of exposure, as described above, and were disrupted by homogenization in a ground-glass homogenizer fitted with a ground-glass pestle and using a buffer consisting of 154 mM NaCl and 10 mM sodium-potassium phosphate (pH 7.4). Aliquots were withdrawn for measurement of DNA (Trauth et al. 2000).

ChAT assays were conducted following published techniques (Lau et al. 1988) using a substrate of 50 μM [^14^C]acetyl–coenzyme A (specific activity, 60 mCi/mmol; PerkinElmer Life Sciences, Boston, MA). Labeled ACh was counted in a liquid scintillation counter and activity calculated as nanomoles synthesized per hour per microgram DNA.

TH activity was measured using [^14^C]tyrosine as a substrate and trapping the evolved ^14^CO_2_ after coupled decarboxylation (Lau et al. 1988; Waymire et al. 1971). Each assay contained 0.5 μCi of generally labeled [^14^C]tyrosine (specific activity, 438 mCi/mmol; Sigma) as substrate, and activity was calculated on the same basis as for ChAT.

### Data analysis

All studies were performed on 8–16 separate cultures for each measure and treatment, using 2–4 separate batches of cells. Results are presented as mean ± SE, with treatment comparisons carried out by analysis of variance (ANOVA) followed by Fisher’s protected least significant difference test for post hoc comparisons of individual treatments. In the initial test, we evaluated two ANOVA factors (treatment, cell batch) and found that the results did not vary among the different batches of cells, so results across the different batches were combined for presentation. Significance was assumed at *p* < 0.05.

## Results

### PFOS

**Figure 1:**
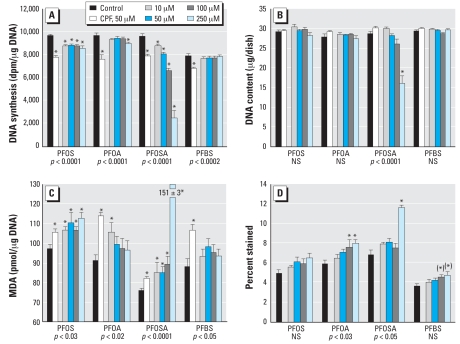
Effects of perfluorinated chemicals in undifferentiated PC12 cells after 24 hr of exposure. (*A*) DNA synthesis. (*B*) DNA content. (*C*) Lipid peroxidation. (*D*) Cell viability evaluated with trypan blue. Abbreviations: dpm, disintegrations per minute; NS, not significant. Values shown are mean ± SE of 8–16 determinations for each group. ANOVA across all treatments in a given experiment is shown at the bottom of each panel. In (*A, B,* and *C*), 50 μM CPF was included as a positive test compound for comparison with the perfluorinated chemicals; CPF does not decrease viability (Song et al. 1998) and was therefore not included in (*D*). *Individual treatments differ significantly from the corresponding control. The asterisks in parentheses for PFBS in (*D*) denote significant differences that were found in the post hoc test despite the absence of a significant overall effect by ANOVA; this was carried out because two-factor ANOVA (treatment, differentiation state) for trypan blue staining in both the undifferentiated state and during differentiation (Figure 3B) indicated a significant main treatment effect (*p* < 0.02) without a treatment × state interaction.

In undifferentiated PC12 cells, PFOS elicited a small but statistically significant reduction in DNA synthesis within 24 hr of exposure (Figure 1A). The effect was smaller than that elicited by 50 μM CPF, even when the PFOS concentration was raised to 250 μM. None of the concentrations elicited a decrement in the number of cells as monitored by DNA content (Figure 1B). Nevertheless, PFOS evoked a greater degree of lipid peroxidation than did CPF (Figure 1C), with significant effects even at the lowest concentration (10 μM). The effects were insufficient to trigger a loss of viability as monitored by trypan blue exclusion (Figure 1D).

**Figure 2:**
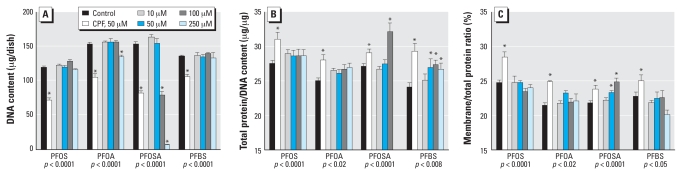
Effects of perfluorinated chemicals on DNA content and protein ratios in differentiating PC12 cells after 6 days of exposure. (*A*) DNA content. (*B*) Total protein/DNA ratio. (*C*) Membrane/total protein ratio. Values shown are mean ± SE of 8–16 determinations for each group. ANOVA across all treatments in a given experiment is shown at the bottom of each panel. For comparison with the perfluorinated chemicals, 50 μM CPF was included as a positive test compound. Determinations for 250 μM PFOSA were not performed in (*B*) and (*C*) because of the small number of cells remaining. *Individual treatments differ significantly from the corresponding control.

**Figure 3:**
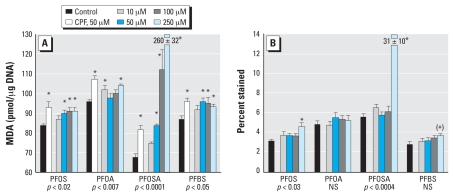
Effects of perfluorinated chemicals on cell damage markers in differentiating PC12 cells after 4 days of exposure. (*A*) Lipid peroxidation assessed by the MDA concentration. (*B*) Trypan blue staining of nonviable cells. NS, not significant. Values shown are mean ± SE of 8–16 determinations for each group. ANOVA across all treatments in a given experiment is shown at the bottom of each panel. For comparison with the perfluorinated compounds, 50 μM CPF was included as a positive test compound in (*A*), but because CPF does not decrease viability (Song et al. 1998), it was not included in (*B*). *Individual treatments differ significantly from the corresponding control. The asterisk in parentheses in (*B*) denotes a significant difference that was found for PFBS in the post hoc test despite the absence of a significant overall effect by ANOVA; this was carried out because two-factor ANOVA (treatment, differentiation state) for trypan blue staining in both the undifferentiated state (Figure 1D) and during differentiation indicated a significant main treatment effect (*p* < 0.02) without a treatment × state interaction.

In differentiating cells, 6 days of exposure to PFOS failed to cause any alterations in indices of cell number (Figure 2A), size (Figure 2B), or the membrane outgrowth associated with neurite formation (Figure 2C), whereas the positive test compound, CPF, showed significant reductions in DNA content and increments in both of the protein ratios. Indices of lipid peroxidation and cell viability were conducted after 4 days of exposure, because these factors represent a forerunner of cell loss. In contrast to the effects in undifferentiated cells, PFOS evoked less oxidative stress than did CPF (Figure 3A). PFOS decreased cell viability only at the highest concentration (Figure 3B).

**Figure 4:**
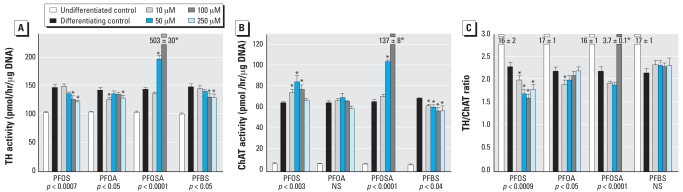
Effects of perfluorinated chemicals on differentiation of PC12 cells into DA and ACh phenotypes, assessed after 6 days of exposure by measuring (*A*) TH activity, (*B*) ChAT activity, and (*C*) TH/ChAT ratio. NS, not significant. Values shown are mean ± SE of 8–16 determinations for each group. To evaluate the extent to which differentiation is affected, we compared determinations in undifferentiated cells. ANOVA across all treatments comparing effects in the differentiating cells only is shown at the bottom of each panel. Determinations for 250 μM PFOSA were not performed because of the small number of cells remaining after 6 days of treatment. *Individual treatments differ significantly from the corresponding control.

With the onset of differentiation, PC12 cells showed increased expression of both TH (Figure 4A) and ChAT (Figure 4B), with a much greater effect on the latter, so the TH/ChAT ratio fell by nearly an order of magnitude (Figure 4C). PFOS interfered with the differentiation into the DA phenotype, as evidenced by a decrement in TH that was significant at concentrations > 50 μM (Figure 4A). At the same time, it enhanced expression of the ACh phenotype, as shown by significant increases in ChAT (Figure 4B); the effect peaked at 50 μM PFOS and then declined, thus displaying an “inverted-U” concentration–effect relationship. The combination of reduced TH and augmented ChAT produced a robust net shift toward the ACh phenotype, as shown by a significant reduction in the TH/ChAT ratio, even at the lowest PFOS concentration (Figure 4C).

### PFOA

Unlike PFOS, 24 hr of exposure of undifferentiated cells to PFOA produced inhibition of DNA synthesis only at 250 μM, the highest concentration tested (Figure 1A). As before, there were no effects on DNA content (Figure 1B). PFOA also produced a significant overall increase in lipid peroxidation, but the effect achieved statistical significance at only one concentration (10 μM); unlike PFOS, the effect was smaller than for the positive test compound, CPF (Figure 1C). Cell viability was significantly reduced at the two highest concentrations (Figure 1D), but the effect was not statistically distinguishable from the nonsignificant increase seen with PFOS (no interaction of treatment × agent in a two-factor ANOVA).

In differentiating cells, PFOA also proved negative for effects on cell number, except at the highest concentration (Figure 2A), and had no discernible impact on the protein/DNA ratio (Figure 2B) or the membrane/total protein ratio (Figure 2C); however, significant effects were seen for all markers with CPF. The differentiating cells also showed some evidence of oxidative stress elicited by PFOA, albeit to a lesser extent than for CPF (Figure 3A), and there were no effects on cell viability as monitored by trypan blue exclusion (Figure 3B). Unlike PFOS, PFOA had only minor effects on differentiation of PC12 cells into the DA and ACh phenotypes. We observed a small decrement in TH activity that was significant at only two of the four concentrations tested (Figure 4A). There was no significant overall effect on ChAT (Figure 4B). The TH/ChAT ratio similarly showed only a small but statistically significant decrement at the lowest PFOA concentration (Figure 4C).

### PFOSA

The effects of PFOSA were substantially different from those of PFOS or PFOA. In undifferentiated cells, PFOSA produced significant inhibition of DNA synthesis at all concentrations tested (Figure 1A). The reduction was equivalent to that of CPF at equimolar concentrations and then showed progressively greater loss at higher concentrations, so that at 250 μM PFOSA, DNA synthesis was almost totally arrested. Even within the span of the 24-hr exposure, 250 μM PFOSA caused a 50% decrease in the number of cells, as monitored by DNA content (Figure 1B). Because this reduction occurred in less than the doubling time for undifferentiated PC12 cells (48–72 hr), it suggested that there was an adverse effect on existing cells rather than just inhibition of new cell formation. Indeed, we found a greater degree of oxidative stress for PFOSA than for CPF, even at one-fifth the concentration, and a massive increase in lipid peroxidation at the highest concentration (Figure 1C). The effects were accompanied by a major decrease in viability, indicated by a rise in trypan blue–stained cells (Figure 1D).

In differentiating cells, 6 days of exposure to PFOSA produced significant decrements in DNA content, with a near-total loss of cells at the highest concentration (Figure 2A); accordingly, protein ratios could not be evaluated at 250 μM. At 100 μM PFOSA, the remaining cells showed a significant increase in the protein/DNA ratio (Figure 2B), and there were small increments in the membrane/total protein ratio that achieved significance at 50 and 100 μM (Figure 2C). Because of the loss of cells at 6 days, we evaluated indices of cell damage at the 4-day point. Lipid peroxidation was readily demonstrable at PFOSA concentrations > 10 μM, with a massive increase at 250 μM (Figure 3A), at which point loss of viability was readily demonstrable (Figure 3B).

Assessments of the impact of PFOSA on neurotransmitter phenotype were likewise truncated at 100 μM since few cells survived for 6 days at 250 μM. PFOSA had a promotional effect on TH at 50 or 100 μM, reaching three times control values at the higher concentration (Figure 4A). Differentiation into the ACh phenotype was also augmented by PFOSA (Figure 4B). However, there was a disparate concentration–effect relationship for the two phenotypes: at low concentrations, PFOSA shifted differentiation toward the ACh phenotype, as evidenced by a decrease in TH/ChAT, whereas at 100 μM, the effect on the DA phenotype predominated, producing a large increment in TH/ChAT (Figure 4C).

### PFBS

The effects of PFBS were somewhat similar to those of PFOA. In undifferentiated cells, there was little or no effect on DNA synthesis (Figure 1A), no shortfall in cell numbers (Figure 1B), and no significant lipid peroxidation (Figure 1C), although at high concentrations there was a small loss of viability (Figure 1D). Similarly, in differentiating cells, PFBS did not evoke a reduction in DNA content (Figure 2A), although it did produce significant cell enlargement as evidenced by an increase in the total protein/DNA ratio (Figure 2B). Like PFOA, PFBS did not change the membrane/total protein ratio (Figure 2C). PFBS evoked lipid peroxidation in differentiating cells, of about the same magnitude as that seen with PFOA but slightly less than that of the positive test compound, CPF (Figure 3A). Viability in differentiating cells was not compromised until the PFBS concentration was raised to 250 μM (Figure 3B). Notably, though, PFBS had a unique effect on differentiation into the two neurotransmitter phenotypes, displaying a concentration-dependent decrease in both the expression of TH (Figure 4A) and ChAT (Figure 4B), a pattern that was not seen with any other agent. Accordingly, the ratio of TH/ChAT was unchanged (Figure 4C) because both enzymes were reduced in parallel by PFBS.

## Discussion

To our knowledge, this is the first demonstration that PFAAs do indeed have direct, developmental neurotoxicant actions and that they target specific events in neural cell differentiation. In general, the rank order of adverse effects was PFOSA > PFOS > PFBS ≈ PFOA. However, superimposed on this scheme, the various agents differed in their underlying mechanisms and specific outcomes, indicating that all PFAAs are not the same in their impact on neurodevelopment and that it is unlikely that there is one simple, shared mechanism by which they all produce their effects.

The greater toxicity of PFOSA can be partially attributed to its less hydrophilic nature. Because the other agents are free acids (PFOA) or sulfonic acids (PFOS, PFBS), PFOSA will more readily cross the cell membrane, achieving higher intracellular levels. By itself, this finding gives important information readily translatable to developmental neurotoxicity *in vivo*: PFAAs that are more hydrophobic or that form less polar metabolites are likely to be more problematic, especially since the same physicochemical properties govern passage across the placental and blood–brain barriers. Nevertheless, pharmacokinetic differences cannot account for the disparities in actions among the various PFAAs: PFOS elicited larger changes than PFBS, despite the fact that both are sulfonic acids, and all four agents had differing, even opposite, actions on neurotransmitter phenotypes. Certainly, one likely possibility for the greater toxicity of PFOSA is its ability to uncouple mitochondrial oxidative function, whereas PFOS and PFOA act simply as surfactants at the mitochondrial membrane (Starkov and Wallace 2002). This feature may contribute to disparities in the targeting of specific events in cell differentiation that are affected by oxidative stress and other downstream events linked to mitochondrial dysfunction.

PFOSA was the only one of the agents tested that matched or exceeded the ability of CPF to inhibit DNA synthesis in undifferentiated cells. Likewise, PFOSA elicited the greatest degree of oxidative stress and cell loss, regardless of whether cells were undifferentiated or differentiating. Nevertheless, even with PFOSA we did not see significant loss of cell viability until the concentration was raised to 250 μM, implying that there are factors other than cytotoxicity that contribute to the net effects. In fact, our results point to strong promotion of the switch from cell replication to cell differentiation, which would also contribute to a reduction in DNA synthesis and in cell numbers. The protein ratios provide support for this interpretation. First, the protein/DNA ratio increased with PFOSA treatment at concentrations below the threshold for cytotoxicity, indicating an increase in cell size rather than the suppression of growth that would be expected from cytotoxic actions. Second, the membrane/total protein ratio also showed a rise with subtoxic PFOSA treatments, indicating augmented membrane complexity, which is commensurate with generation of neurites and cellular organelles that accompanies differentiation. The final proof of a prodifferentiation effect can be seen from the strong promotion of both neurotransmitter phenotypes, evidenced by marked increases in both TH and ChAT. The differentiation pattern triggered by PFOSA is not, however, a normal one: At low concentrations, the TH/ChAT ratio was slightly, but significantly decreased, whereas at high concentrations, it rose markedly. This means that PFOSA alters the differentiation fate of the cells, switching them weakly to the ACh phenotype at low concentrations, and strongly to the DA phenotype at high concentrations. If similar effects happen *in vivo*, it might be expected that neurons will differentiate into inappropriate phenotypes; this would lead to miswiring of neural circuits, with presynaptic projections for a given neurotransmitter juxtaposed to post-synaptic elements containing incorrect receptors, resulting in nonfunctional synapses. Given the biphasic dose–response curve, the outcomes would be very different for an individual exposed to low doses that promote the ACh phenotype as opposed to an individual receiving higher exposures that promote the DA phenotype. Such switching has already been seen with other agents that produce phenotype shifts in the PC12 model, notably CPF (Barone et al. 2000; Dam et al. 1999; Jameson et al. 2006b; Pope 1999; Rice and Barone 2000; Slotkin 2004a; Vidair 2004). The biphasic nature of the concentration–effect relationship suggests that there are at least two separate mechanisms operating to turn on expression of neurotransmitter phenotypes. Oxidative stress, which was prominent for PFOSA, is likely to be one contributory factor, since oxidative stress itself is a prodifferentiation signal (Katoh et al. 1997). However, it is also possible that PFOSA switches the phenotypes by turning specific DA- or ACh-related genes on or off inappropriately, as has been shown for the organophosphates (Slotkin and Seidler 2007).

Although PFOS also inhibited DNA synthesis in undifferentiated PC12 cells, its effects were less than those seen with the positive test compound (CPF) and far smaller than those seen with PFOSA. There was no parallel reduction in DNA content, which is not surprising given the small effect on DNA synthesis and the fact that a 24-hr exposure is less than a doubling time for PC12 cell replication. Notably, we did not find any reductions in cell numbers even with a 6-day exposure of differentiating cells, showing that the effects of PFOS are indeed fundamentally different from those of PFOSA. This was further confirmed by a lack of any evidence for a global prodifferentiation effect because there were no changes in the protein ratios and only a small degree of oxidative stress. Nonetheless, PFOS altered the phenotypic fate of the cells, promoting the ACh phenotype at the expense of the DA phenotype; the effect was again biphasic, as the increase in ChAT regressed back to normal as the PFOS concentration was raised above the point where lipid peroxidation and cytotoxicity emerged. There are two important points made by these findings: *a*) The impact of PFOS on neurotransmitter phenotype is radically different from that of PFOSA; and *b*) some of the effects on differentiation are distinct from those related to oxidative stress and cytotoxicity, and occupy a part of the concentration–effect curve below the thresholds for those common adverse events.

Of the four agents, PFOA and PFBS had the least effect on most of the parameters connoting cell acquisition and growth. Neither agent produced any substantial inhibition of DNA synthesis or cell loss in undifferentiated cells, and a small decrease in DNA in differentiating cells was found only at the highest concentration, and only for PFOA. Although both agents produced small but significant decreases in viability at concentrations of 100 or 250 μM, the effect was obviously insufficient to have any impact on the total number of cells remaining in the culture. Similarly, PFOA had no effect on protein ratios, and PFBS had only a small effect on total protein/DNA without any impact on membrane/total protein. For phenotypic outcomes, the two agents were substantially different from PFOSA and PFOS, both in the type and magnitude of effects. PFOA caused a slight reduction in TH and, consequently, a minor shift favoring the ACh phenotype (reduced TH/ChAT ratio). In contrast, PFBS retarded differentiation into both phenotypes, an effect not seen with any other agent; accordingly, although the TH/ChAT ratio was unaffected by PFBS, both TH and ChAT were significantly reduced, indicating a likelihood of impaired function for both neurotransmitters. Once again, this demonstrates that the effect on phenotypic fate of the cells is distinct from any other effects on cell replication, growth, or viability.

Our results thus point to the potential for PFAAs to evoke developmental neurotoxicity through direct actions on replicating and differentiating neurons, effects distinct from the indirect consequences of endocrine disruption or metabolic or other secondary mechanisms. Importantly, the various PFAAs differ not only in their propensity to elicit outright neurotoxicity or oxidative stress but also in their ability to augment or suppress specific neurotransmitter phenotypes, thus shifting the differentiation fate of the neuron. These effects can even be in opposite directions for the various PFAAs, and because the concentration–effect curve is biphasic, different levels of exposure can be expected to produce disparate outcomes directed toward a given neurotransmitter phenotype. The fact that there are stark differences in developmental neurotoxicant actions among otherwise similar PFAAs means that it is important to take developmental neurotoxicity into account in the design of future members of this class. As demonstrated here, this can be aided by the use of *in vitro* models that permit rapid and cost-effective screening for developmental neurotoxicity.
